# Notes and News

**Published:** 1899-11-18

**Authors:** 


### Notes and News.

(Collected and Communicated.)

A cottage hospital in commemoration of the Diamond Jubilee has been opened at Castle Douglas. Two general wards and one private ward have been provided.

The Lord Mayor has lent the Mansion House, and will preside at a reading of "The Christmas Carol" by Sir Squire Bancroft, in aid of the Earlswood Asylum, on the afternoon of Friday, December 15th.

At an inquest held a few days ago at Mile End a curious case of transposition of the viscera was described by Dr. Harley, assistant medical officer at the Mile End Infirmary. The inquest was held on the body of a dock labourer, and at the post-mortem examination all the viscera, the lungs, heart, stomach, bowels, liver, and spleen, were found to be transposed. According to the daily papers even the kidneys were transposed.

The Board of Education in Chicago has appropriated 20,000 dols. for this year, to be paid out of the School Fund, for the purpose of providing an official medical inspection of all children who have been absent from school for four days or more. Fifty physicians will be employed in the work. It is hoped that by this means some check will be placed upon the increased prevalence of scarlet fever and diphtheria, which always shows itself on the opening of the schools after the autumn holidays.

Vast as is the system of main drainage by which the sewage of London is carried out to Barking and Crossness, the growth of London has outrun it, and now it is found insufficient for its purpose. The whole subject has recently been under the consideration of the Main Drainage Committee of the County Council, who, after taking into consideration the circumstances under which the system was established, and the work which it now has to perform, have come to the conclusion that the time has arrived when a very heavy expenditure must be incurred in providing more sewer accommodation. The recommendation, if carried out in its entirety, would involve a capital expenditure of about £3,000,000. At present, however, it is only proposed to proceed with the most pressing works. It is especially recommended that the capacity of the outfall sewers should be increased without delay, at a cost of about £1,250,000.

Her Majesty the Queen, accompanied by Princess Christian, Princess Henry of Battenberg, and the Duke of Connaught, visited Bristol on Wednesday for the purpose of opening the new Convalescent Home for the city. Fully six miles of the route were decorated, and a large number of bands and detachments of troops were present. The weather was fine, and the ceremony was successfully performed amidst much enthusiasm.

On Tuesday afternoon the Hospital for Diseases of the Throat, Nose, and Ear, in Golden Square, was re-opened by Lady Rothschild, after undergoing reconstruction and being considerably enlarged at a cost of about £15,000. The present extension has doubled the accommodation provided for patients. In addition to the two new wards, containing 50 beds for in-patients, a waiting-room and a consulting-room for out-patients have been added, as well as a central staircase and lifts.

The outbreak of typhoid fever in the constabulary barracks in Dublin, to which we referred some time ago, has been traced to the milk supply. A dairyman from whose farm milk was supplied over a considerable area was attacked in August with an illness which turned out to be typhoid fever. His sister also was attacked. In the course of September 32 cases occurred in the constabulary barracks, which, however, did not express the whole extent of the outbreak, other people unconnected with the barracks also being affected.

The Society of Members of the Royal College of Surgeons of England, finding that the Council of the College have taken no notice of the memorial presented to them in regard to the enlargement of the proposed Charter beyond informing them that they consider it inopportune, have forwarded a statement of their case against the Council of the College to the Lord President of the Privy Council. In this they respectfully urge that his Lordship will, on grounds of public policy, either refer this Supplementary Charter back to the Council of the College in order that provision may be made for the representation of members, or that, before granting any Charter, he will hear the Society of Members by counsel.

The secretary of St. John Ambulance Association communicates the following intimation: "It has been notified by the Central British Red Cross Committee that applications for employment under the Red Cross, and offers of ambulance material, have been so varied and numerous that it is desired to make it generally known that for the present no further applications for employment can be entertained, and no further contributions of ambulance material can be received, except those already arranged for." Money contributions for the general purposes of the Central British Red Cross Committee are much needed. All remittances should be made payable to the St. John Ambulance Association, and should be crossed "London and Westminster Bank." Viscount Knutsford and the Central Executive Committee of the St. John Ambulance Association offer their most grateful thanks to all those who have contributed, or have offered to contribute, and say that nothing but the fact that the supply of material has been far in excess of requirements prevents them availing themselves of the hundreds of kind offers which have been daily received.

